# Premature mortality due to cervical cancer: study of interrupted time series

**DOI:** 10.11606/s1518-8787.2020054002528

**Published:** 2020-11-27

**Authors:** Maria Isabel do Nascimento, Felipe Corrêa Massahud, Nathália Giácomo Barbosa, Cássio Destefani Lopes, Vanessa da Costa Rodrigues

**Affiliations:** I Universidade Federal Fluminense Faculdade de Medicina NiteróiRJ Brasil Universidade Federal Fluminense. Faculdade de Medicina. Mestrado Profissional em Saúde Materno Infantil. Niterói, RJ, Brasil; II Universidade Federal Fluminense Faculdade de Medicina NiteróiRJ Brasil Universidade Federal Fluminense. Faculdade de Medicina. Niterói, RJ, Brasil

**Keywords:** Public Policy, Noncommunicable Diseases, Uterine Cervical Neoplasms, Mortality, Premature, Interrupted Time Series Analysis

## Abstract

**OBJECTIVE:**

To verify the effect of the Pact for Health on premature mortality (30–69 years) attributed to cervical cancer in Brazil and its macroregions, using interrupted time series analysis.

**METHODS:**

Segmented regression was used to assess “change in level” and “change in trend” in premature mortality rates attributed to cervical cancer considering the post-Pact period (2010-2018), controlling by the pre-Pact period (1998–2006). Understanding the triennium 2007-2009 as essential for the adoption and implementation of the policy, it was excluded from the main modeling, but assessed in the sensitivity analysis.

**RESULTS:**

From 1998 to 2018, there were more than 119,000 deaths due to cervical cancer in women aged 30 to 69 years in Brazil. The Northern region experienced the highest rates (> 20 per 100,000). Comparing with baseline (1998–2006), segmented regression showed a progressive increase in changing trend from cervical cancer deaths in Brazil as a whole (coefficient = 0.513; 95%CI 0.430 to 0.596) and in the Southeast region (coefficient = 0.515; 95%CI 0.358 to 0.674), South region (coefficient = 0.925; 95%CI 0.642 to 1.208), and Midwest region (coefficient = 0.590; 95%CI 0.103 to 1.077). The Northeast region presented the most promising effects with immediate reduction in change level (-0.635; 95%CI −1.177 to −0.092) and progressive reduction in the changing trend of premature deaths (coefficient= −0.151; 95%CI −0.231 to −0.007).

**CONCLUSIONS:**

Premature mortality rates due to cervical cancer are high in Brazil and its macroregions. This interrupted time series was not able to reveal the effectiveness of initiatives related to the Pact for Health on premature deaths from cervical cancer nationally and in all macroregions equally. The best results are restricted to the Northeast region.

## INTRODUCTION

Premature death occurs at the age of 30 to 69 years and reaches about 15 million people, every year, in the world. Actions to reduce this number focus mainly on non-communicable diseases (NCD), such as cancer. International entities have prepared documents proposing strategies and goals to contain such outcomes ^1^ .

Cervical cancer (CC) is a completely preventable neoplasm but continues to contribute to the global burden of diseases. As a cause of premature deaths measured by Disability-Adjusted Life Year (DALY), the disease exceeds breast cancer in 23 countries located in sub-Saharan Africa and parts of South and Central America ^2^ . Historical series of ten consecutive years showed that mortality rates increased in 8 out of 11 countries in Central Asia, in women under 50 years of age ^3^ . In Brazil, information provided by the Department of Informatics of the Unified Health System (Datasus) ^4^ shows the occurrence of 6,526 deaths due to CC in 2018, with the premature age group (30–69 years) accounting for 74.0% of deaths. The highest frequency of deaths occurred between 50 and 59 years.

The Pact for Health ^5^ is a public policy, established in 2006, which is part of policies to support the Unified Health System (SUS). It is supported by three pillars (Pact for Life, Pact in Defense of SUS and Management Pact) that aim to optimize intergovernmental relations for better articulation and cooperation between federative entities. It is understood as fundamental to the strengthening of comprehensive health care, with the provision and utilization of the resources organized by levels of care ^6^ and regionalization of health services ^7^ .

Although, in 2011, the intergovernmental relationship model for consolidation of SUS has evolved into commitments signed in contract (Organizational Contract for Public Action — COAP), the CC was prioritized among the primary indicators of the Pact and remained a target of health authorities' actions in subsequent versions. Additionally, the goal of reducing premature deaths related to NCD, which include malignant neoplasms, was also included in the list of guidelines, objectives, goals and indicators ^8^ . Two indicators (ratio of cytopathological tests and proportion of follow-up/treatment of high-grade intraepithelial lesion) that were prioritized by the Pact targeted the CC control, and technical notes and results are available in Datasus ^4^ . Cancer screening and early detection procedures followed by effective treatments improve the approach of disease that is detected in earlier clinical stages and reflecting on the reduction in mortality ^9^ . In this way, it was postulated that the measurement of mortality due to CC can provide an insight into the effectiveness of the actions induced by the Pact for Health in Brazil.

Considering that public policy evaluations may contribute to identifying their impact and, if necessary, suggest the review of their actions ^10^ , and considering there is a scarcity of studies monitoring the indicators related to the Pact for Health, this study aimed to assess trends in premature mortality rates due to cervical cancer after the implementation of the Pact for Health and policies derived from it, controlling by the period before implementation of the pact in Brazil and its macroregions.

## METHODS

Assuming that the goals and commitments prioritized in the policies related to the Pact for Health can have reflection on the reduction in premature and preventable deaths in several contexts, including CC, this ecological study was developed using interrupted time series (ITS) to verify whether the introduction of the Pact for Health has reduced premature deaths due to CC in Brazil and its macroregions. The analysis considered the dynamics of the adherence to the Pact, because, according to Technical Note No. 08/2012 ^11^ , 18.0% of Brazilian municipalities did not immediately adhere to the policy. Thus, for the composition and analysis of the historical series, the periods 1998-2006 were defined as “pre-Pact” and 2010-2018 as “post-Pact.” The years 2007, 2008 and 2009 comprised the phase of the adoption and implementation of the Pact for Health.

Datasus ^a^ website was consulted to obtain information on deaths that occurred in the five-year age groups, from 30 to 69 years, following the steps: Health Information, Vital Statistics, Mortality — 1996 to 2018, by ICD-10, General Mortality, Geographic Coverage: Brazil by Region and Federation Unit.

The basic causes of death coded according to the 10th Revision of the International Statistical Classification of Diseases and Related Health Problems (ICD-10) were considered, seeking codes C53 (malignant neoplasm of cervix uteri), C54 (malignant neoplasm of corpus uteri) and C55 (malignant neoplasm of the uterus, part unspecified), for subsequent redistribution.

The correction of the number of deaths due to CC was made following two steps. First, the proportional redistribution of deaths coded as C55 (uterus, part unspecified) was performed, as recommended by Antunes and Wünsch-Filho ^12^ ; to do so, deaths coded as C55 were proportionally relocated as C53 and C54. The second step aimed to correct deaths recorded as ill-defined underlying causes (ICD-10 R00 to R99). In this step, the procedure ^13^ used determines a percentage to be applied to deaths in each unit of analysis, by age group and unit of time. The mathematical equations used in this stage can be found in a study that corrected deaths caused by breast cancer ^14^ .

The population of women by age group of premature death (30 to 69 years) was obtained from IBGE ^15^ sites (population projection from 2000 to 2060) and Datasus ^4^ (population 1998 and 1999). The quantitative ones were organized into eight five-year ranges (30–34, 35–39, 40–44, 45–49, 50–54, 55–59, 60–64, 65–69 years).

To verify the strength with which mortality due to CC affects the population of women in Brazil and its macroregions, age-specific mortality rates for each age group and year were first estimated. The corrected number of deaths composed the numerator, and the projected population, according to age group and year, composed the denominator. Next, the rates were standardized by the direct method, using the world population as the standard ^16^ . The results were computed by 100,000 women-year.

### Interrupted Time Series (ITS) Analysis

ITS modeling and segmented regression ^17^^,^^18^ were implemented to verify the trend of premature deaths caused by CC after the introduction of the Pact for Health. Two new components – labeled “change in level” and “change in trend” – were introduced into the analysis, along with dependent variable (mortality rate), year (1998 to 2018) and time (sequence 1 to 21). To examine the change in level, the digit 0 was allocated to the “pre-Pact” period and 1 to “post-Pact.” To verify the change in trend, the “pre-Pact” period was considered as 0, and the digits 1, 2, 3… were subsequently assigned to each time frame of the “post-Pact” period. Thus, in addition to dependent variable (mortality rates), the variables time, change in level and change in trend entered the segmented regression.

Anticipating issues that are inherent to parametric analyses, we verified the existence of correlation of residues for *n* lags, through the Durbin-Watson statistics and its critical values ^19^ . This method allows the interpretation of values around 2 as absence of significant serial correlation ^17^ and *p* value with alpha level < 0.05 as suggestive of autocorrelated residuals. Finally, the inspection of autocorrelation function (ACF) and partial autocorrelation function (PACF) graphs was performed to visually explore the existence and number of possible lags involved in the autoregressive process. The modeling was conducted considering parsimony criteria and quality of adjustment provided by Akaike information criterion (AIC), whose lower value suggests better quality of adjustment. Parameter estimation was performed via generalized linear models using the *gls* command and the RStudio platform.

### Sensitivity Analysis

Sensitivity analysis was conducted implementing segmented regression without excluding the three-year implementation of the Pact for Health (with all years). The modeling considered only two segments, with the triennial of implementation first added to the final period, composing the pre-Pact (1998-2006) and post-Pact (2007-2018) periods. In the subsequent modeling, the triennial of implementation period was moved again, and the segments were defined as pre-Pact (1998-2009) and post-Pact (2010-2018).

### Ethical Procedures

The study follows the recommendations for scientific research involving human beings and was conducted with the use of secondary information made available online by Datasus, and, therefore, it is exempt from formal ethical procedures.

## RESULTS

**Figure 1:**
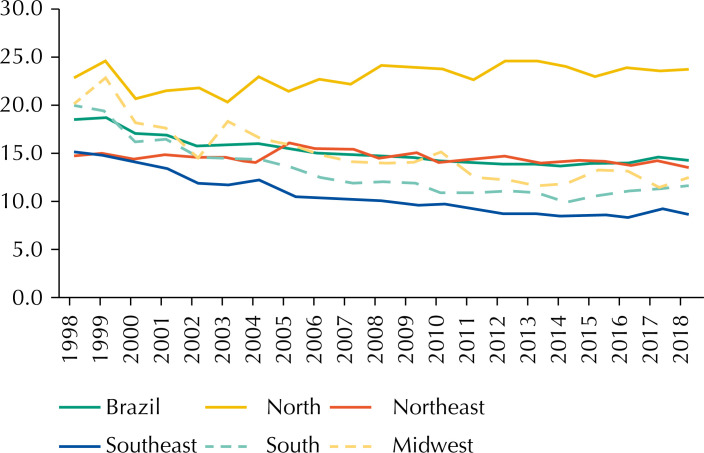
Simple trends in cervical cancer premature mortality rates, standardized by age (30-69 years) in Brazil and in its macroregions, per 100,000 women, from 1998 to 2018.

From 1998 to 2018 there were 102,850 deaths from CC (C53) in Brazil, and 77,101 (75.0%) occurred in the premature age group (30 to 69 years). The number of deaths increased by 52.0% and 55.2% after corrections by uterus, part unspecified, and by uterus, part unspecified together with ill-defined causes, respectively. The highest proportion of deaths occurred in the Southeast (37.7%), followed by the Northeast (28.0%), South (16.0%), North (10.7%) and Midwest (7.6%). Overall, premature mortality rates by age-standardized CC were high. Only the Southeast (as of 2009) and South (in 2014) regions outlined estimates lower than 10 per 100,000 ( Figure 1 ).

The results of the segmented regression that compared the post-Pact (2010–2018) and reference (1998–2006) periods are summarized in the Table . Initial exploratory models were estimated, assuming total independence of the data, as well final models, corrected by the insertion of *n* terms related to serial autocorrelation, as specified by the partial autocorrelation function.

**Table:** Effect of the introduction of the Pact for Health on premature mortality rates from cervical cancer in Brazil and in its macroregions: changes in level and change in trends and parameters estimated by interrupted time series analysis and by comparing the periods 2010–2018 and 1998–2006.

Final behavior of mortality after intervention			Initial exploratory model with no serial autocorrelation terms				Final model with n terms of serial autocorrelation			
Regressors		Interpretation	Coefficient	95%CI	DW Statistic a	AIC	Coefficient	95%CI	AIC b	Terms AR c
Brazil										
	Change in level	Abrupt elevation	0.48	-0.467 to 1.428	0.086	36.772	0.497	0.039 to 0.955	15.918	p = 3
	Change in trend	Progressive elevation	0.495	0.351 to 0.638			0.513	0.430 to 0.596		
	Intercept		18.872	18.301 to 19.443			18.821	18.518 to 19.124		
	Time		-0.460	-0.561 to −0.358			-0.467	-0.523 to −0.411		
North										
	Change in level	Abrupt elevation	2.522	-0.086 to 5.131	NS	65.065	2.629	0.422 to 4.837	61.234	p = 1
	Change in trend	Not detected	0.118	-0.276 to 0.513			0.128	-0.207 to 0.464		
	Intercept		22.703	21.132 to 24.274			22.725	21.399 to 24.052		
	Time		-0.122	0.402 to 0.156			−0.133	0.371 to 0.104		
Northeast										
	Change in level	Abrupt reduction	-1.062	-2.149 to 0.025	0.018	40.561	-0.635	-1.177 to −0.092	26.596	p = 2
	Change in trend	Progressive reduction	-0.142	-0.307 to 0.022			-0.151	-0.231 to −0.071		
	Intercept		14.494	13.839 to 15.149			14.516	14.201 to 14.833		
	Time		0.079	0.036 to 0.196			0.058	0.0007 to 0.116		
Southeast										
	Change in level	Not detected	0.881	-0.122 to 1.886	0.098	38.336	0.889	-0.156 to 1.934	27.669	p = 1
	Change in trend	Progressive elevation	0.517	0.365 to 0.669			0.516	0.358 to 0.674		
	Intercept		15.754	15.149 to 16.359			15.753	15.123 to 16.383		
	Time		-0.609	-0.716 to −0.502			-0.609	-0.721 to −0.497		
South										
	Change in level	Not detected	1.051	-0.668 to 2.770	0.098	53.394	1.059	-0.821 to 2.940	46.765	p = 1
	Change in trend	Progressive elevation	0.918	0.657 to 1.178			0.925	0.642 to 1.208		
	Intercept		20.091	19.056 to 21.127			20.131	18.995 to 21.265		
	Time		-0.869	-1.053 to −0.685			-0.873	-1.073 to −0.673		
Midwest										
	Change in level	Not detected	0.994	-2.624 to 4.613	NS	74.232	0.933	-2.271 to 4.137	73.43	p = 1
	Change in trend	Progressive elevation	0.565	0.017 to 1.112			0.59	0.103 to 1.077		
	Intercept		21.283	19.104 to 23.463			21.333	19.407 to 23.260		
	Time		-0.734	-1.121 to −0.346			-0.743	-1.087 to −0.398		

95%CI: 95% confidence interval.

a Durbin Watson statistic (p value < 0.05 suggests serial autocorrelation).

b Akaike Information Criterion (smaller values suggest better quality of fit).

c Terms of autocorrelation defined by the function of autocorrelation and partial autocorrelation.

The two modeling processes captured quite convergent results. Specifically, there was a change in trend, with an increase in premature mortality due to CC in Brazil and in the Southeast, South and Midwest macroregions and stability in the North region, comparing the periods 2010-2018 and 1998–2006. Only the final model captured a trend of decline in death rates in the Northeast region. A particular finding observed in the North and Northeast regions was ta statistically significant change in the level of premature mortality due to CC — which followed the introduction of the Pact in Brazil. This occurred only in the process of estimating parameters of the final models, with the insertion of serial autocorrelation terms. Although the direct contribution of these findings is not so obvious when it comes to public policies, they are essential to predict the impact of interventions considering predicted and counterfactual estimates. Taking as an example Brazil as a whole and the equation “Result_jt_= βo + β_1_ * time_t_ + β_2_ * level_j_ + β_3_ * trend_jt_ + e_jt_,” predicted values (20.042) and counterfactual values (10.415) were estimated, verifying that the average rate of premature deaths due to CC in 2018 would approach 7.38 per 100,000 if the initiatives that occurred in the pre-Pact phase followed in matching to current policies ( Table ).

**Figure 2:**
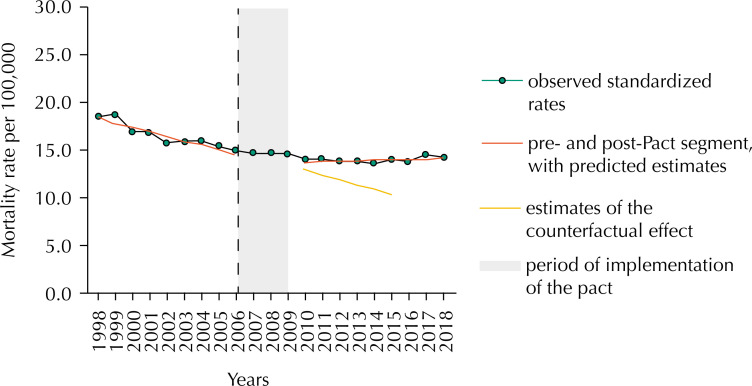
Effect of the introduction of the Pact for Health on premature mortality rates due to cervical cancer in Brazil, considering the periods 1998–2006 (pre-Pact), 2007–2009 (introduction of the Pact) and 2010–2018 (post-Pact).

The pictorial representation of the rates relative to Brazil shows that, while baseline coefficients (1998–2006) experienced visible and prolonged decline in counterfactual effect, the post-Pact segment outlines mortality adjusted in the opposite direction, statistically detected by slope coefficients (change in level: 0.497, 95%CI 0.039–0.955; and change in trend: 0.513, 95%CI 0.430–0.596) ( Figure 2 ).

**Figure 3:**
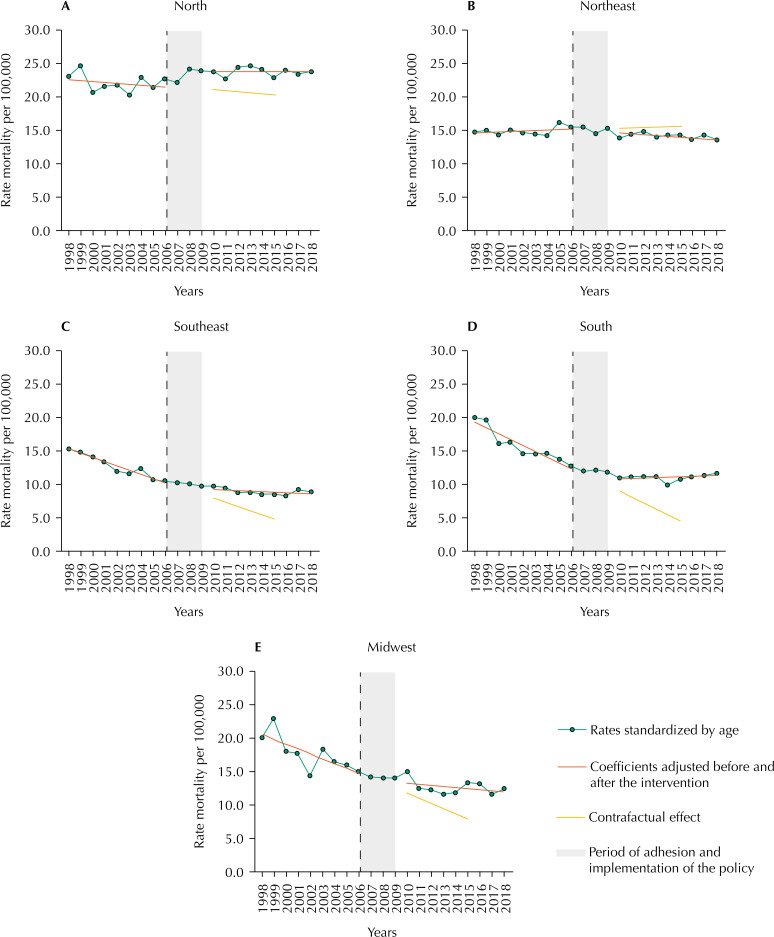
Effect of the introduction of the Pact for Health on premature mortality rates due to cervical cancer in Brazilian macroregions, considering the periods 1998–2006 (pre-Pact), 2007–2009 (introduction of the Pact) and 2010–2018 (post-Pact).

The set of graphs that describes the macroregions facilitates visual comparison among them ( Figure 3 ). The most promising scenario comes from the Northeast region. Although the statistically significant reductions in the level and trend are still discrete and not noticeable visually, they are findings that strengthen the actions and policies in force. The fact that the tracing of the counterfactual effect observed from the baseline estimates follows clearly in increasing gives indications of the magnitude of the rates that the Northeast region could experience in the absence of gains enabled by the introduction of the policies of the Pact. As to the other regions, in addition to the abrupt increase in the level being captured by the segmented regression, the North region outlined the worst results, mainly due to the magnitude of the values predicted and observed, because no change in trend was delineated. As for the more developed macroregions of the country (Southeast, South and Midwest), the graphs also suggest that the Pact and the policies derived from it have not had an impact on the premature deaths attributed to CC ( Figure 3 ).

Sensitivity analysis did not show divergences in relation to the main results. Overall, the sensitivity analysis captured changes in trend in the same direction, both in the interruption in 2006 and in 2010. The exception was the segmented series in 2010 of the Northeast region, which did not capture the progressive decline of the trend previously detected. The change in level observed in the North and Northeast was not captured at any of the two cutoff points.

## DISCUSSION

When analyzing premature mortality rates related to CC, a challenging scenario was found to control this neoplasm in Brazil. The standardized coefficients had systematically high values, above 10 deaths per 100,000 women, except in the Southeast region. In addition, the segmented regression showed that the Pact for Health and its policies have not yet had nationally favorable effects on CC control measures and are captured by indicators of premature mortality.

The Pact introduced a novelty in health management in Brazil, since it aimed to generate results and achieve goals outlined by the population's health needs. The responsibilities among the entities were signed by the term of management commitment, a document that lasted from 2006 to 2012, when the migration to the new format based on COAP ^20^ occurred. In a quasi-experimental assessment, Krott and Guimarães ^20^ showed that in the fourth year of municipal-level agreement, positive results related to primary care begin to appear. These findings show the property of excluding from the analysis the periods to accept and to start the policy implementation.

The elimination of CC has become a world priority ^21^ , and it is appropriate to implement assessments that can contribute to the understanding of the real scenario. Although premature mortality from CC is not clearly specified in the Pact's list of indicators, the objectives, guidelines and targets contained therein aim to improve the health conditions of the population in general, making its control a priority and reducing premature deaths caused by cancer ^11^ . In the context of CC, the objectives of the policy initially aimed to: (i) increase coverage to 80% of the preventive examination of the CC, according to the 2006 protocol; and (ii) give incentives to see and treat precancerous lesions, with as little damage as possible and at the outpatient level. The indicators of the Pact selected for coverage (ratio of cytopathological tests of CC in women aged 25 to 64 years and the population of the same age group) and for treatment (percentage of follow-up and treatment of high-grade lesions in the cervix) were well below target ^4^ between 2006 and 2012. In addition, ^22^ more recent studies that estimated the annual need for tests and procedures in women of the target age of CC (25–64 years) pointed to the deficit of all essential procedures, which ranged from 7% in colposcopies to 74% in type 3 excisions.

Given this adverse scenario, the results of our study related to macroregions also point to a reality of little effectiveness and great challenges. Even the most developed regions (Southeast and South) suffer from indicators of premature mortality from CC in elevation, opposing the post-Pact phase with the baseline period. Simple trend studies, with data on 21st century deaths and broader age groups, have pointed to the reduction in mortality, although this is not occurring homogeneously in all age groups ^23^ and in all the large regions of the country ^23^ ; despite efforts, they tend to increase in some federative units ^24^^,^^25^ .

In general, results of monitoring studies can induce the review of ongoing policies and assist in the planning of new initiatives, as well as in decision-making by managers and health authorities. For example, in the United States, the search for the optimization of resources aimed at reducing premature deaths due to CC led to the inclusion of monitoring indicators designated to measure the capture of priority population ^26^ . We established that at least 20% of women newly recruited for CC tests should be in the condition of rarely or never screened.

To operationalize the analysis of the effect of the Pact for Health on the trend of premature mortality due to CC, this study was developed using ITS and segmented regression analysis ^17^ . These techniques are understood as quasi-experimental, appropriate for longitudinal assessments of clinical interventions ^17^^,^^27^ and in the field of public health ^18^ . Its application in scientific research is timely in Brazil, considering that the country has a broad health information system, with storage of routine data of a reasonable period of time, besides implementing several public policies aimed at improving the health of the population that may be more effective if submitted to concomitant assessments.

A tutorial ^18^ details the step-by-step implementation of the ITS, emphasizing the clear definition (i) of the intervention, (ii) the measure/s of the result/s, (iii) of the pre- and post-intervention periods, as well as (iv) the intervention implementation period. To produce robust results, Bernal et al. ^18^ recommend attention to potential confounding factors and assumptions related to parametric analyses.

In the context of potential confounding factors, which may interfere in the post-implementation period of the policy, attention is drawn to Law No. 12,732, about the 60 days of cancer treatment ^28^ . As the law was regulated in 2012, it could already reflect a reduction in mortality rates from CC, a fact not yet observed in this study. On the other hand, the counterfactual effect ( Figure 2 and 3 ), delineated from coefficients adjusted since the beginning of the series, shows a downward trend in premature deaths due to CC. These results possibly reflect the major CC prevention campaigns implemented by the Viva Mulher (Long Live Women) Program ^29^ .

This study has some limitations. First, the presence of autocorrelation of residues may overestimate the measurements. Given this, the estimation of the models was diversified, and self-regressive terms were introduced with a lag different from 1. Although estimates have converged on similar results, a rational recommendation would be to examine such trends in smaller clusters (federative units and municipalities) and verify what is actually occurring in terms of premature deaths from CC in recent years.

Another issue is that the implementation of public policies happens gradually, and, as expected, part of the Brazilian municipalities did not immediately adhere to the Pact for Health. Another recommendation is to consider a transition period for policy implementation ^18^ . Therefore, the years 2007, 2008 and 2009 were excluded from the main analyses, thus believing that this overview of the effect of the Pact on the behavior of premature deaths attributed to CC is capable of inducing the development of new studies, including appropriate control groups.

In conclusion, this study showed that premature mortality from CC is high in Brazil and its macroregions. This interrupted time series was not able to show the effectiveness of initiatives related to the Pact for Health on premature deaths from cervical cancer nationally and in all macroregions equally. At a time when the elimination of CC is sought in the world, the overview provided by premature mortality until 2018 suggests the need to improve the effectiveness of measures to identify women who are truly at risk of developing CC and prevent deaths of relatively young women in Brazil.

## Footnotes

a Ministry of Health. Department of Informatics of the Brazilian National Health System — DATASUS. Access on Mar 31, 2020. Available from: http://www2.datasus.gov.br/DATASUS/index.php?area=060701 .
