# Supplementary material for: Premature mortality due to cervical cancer: study of interrupted time series
Source: Rev Saude Publica. 2020 Nov 27;54:139. doi: 10.11606/s1518-8787.2020054002528 (PMC7703528; doi:10.11606/s1518-8787.2020054002528)
Supplement: Translation

# Translation: Mortalidade prematura por câncer de colo uterino: estudo de séries temporais interrompidas

**Authors:** Maria Isabel do Nascimento, Felipe Corrêa Massahud, Nathália Giácomo Barbosa, Cássio Destefani Lopes, Vanessa da Costa Rodrigues

**Affiliations:** I NiteróiRJ Brasil Universidade Federal Fluminense. Faculdade de Medicina. Mestrado Profissional em Saúde Materno Infantil. Niterói, RJ, Brasil; II NiteróiRJ Brasil Universidade Federal Fluminense. Faculdade de Medicina. Niterói, RJ, Brasil

**Keywords:** Política Pública, Doenças não Transmissíveis, Neoplasias do Colo do Útero, Mortalidade Prematura, Análise de Séries Temporais Interrompida

## Abstract

**OBJETIVO:**

Verificar o efeito do Pacto Pela Saúde na mortalidade prematura (30–69 anos) atribuída a câncer de colo uterino no Brasil e nas suas macrorregiões, utilizando modelagem de séries temporais interrompidas.

**MÉTODOS:**

A regressão segmentada foi usada para avaliar “mudança de nível” e “mudança de tendência” das taxas de mortes prematuras por câncer de colo uterino no período pós-Pacto (2010–2018), controlando pelo período pré-Pacto (1998–2006). Entendendo o triênio 2007–2009 como essencial para adesão e implantação da política ele foi excluído da modelagem principal, mas avaliado na análise de sensibilidade.

**RESULTADOS:**

De 1998 a 2018, houve mais de 119 mil óbitos por câncer de colo uterino, em mulheres de 30 a 69 anos, no Brasil. A região Norte experimentou as taxas mais altas (> 20 por 100 mil). Comparando à linha de base (1998–2006), a regressão segmentada mostrou progressiva elevação das mortes por câncer de colo uterino no Brasil como um todo (coeficiente angular = 0,513; IC95% 0,430 a 0,596) e nas regiões Sudeste (coeficiente = 0,515; IC95% 0,358 a 0,674), Sul (coeficiente = 0,925; IC95% 0,642 a 1,208), e Centro-Oeste (coeficiente = 0,590; IC95% 0,103 a 1,077). A região Nordeste apresentou os efeitos mais promissores com redução imediata no nível (-0,635; IC95% −1,177 a −0,092) e redução progressiva na tendência de mortes prematuras (coeficiente= −0,151; IC95% −0,231 a −0,007).

**CONCLUSÕES:**

As taxas de mortalidade prematuras por câncer de colo uterino são altas no Brasil e nas suas macrorregiões. Esta série temporal interrompida não foi capaz de revelar efetividade das iniciativas relacionadas ao Pacto pela Saúde sobre as mortes prematuras por câncer de colo uterino nacionalmente e tampouco em todas as macrorregiões igualmente. Os melhores resultados estão restritos à região Nordeste.

## INTRODUÇÃO

Morte prematura ocorre na idade de 30 a 69 anos e atinge cerca de 15 milhões de pessoas, a cada ano, no mundo. As ações para reduzir esse número focam principalmente em doenças crônicas não transmissíveis (DCNT), como o câncer. Entidades internacionais têm elaborado documentos propondo estratégias e metas para conter tais desfechos ^1^ .

O câncer de colo uterino (CCU) é uma neoplasia totalmente evitável, mas continua contribuindo para a carga mundial de doenças. Como causa de mortes prematuras medida por anos de vida perdidos, a doença supera o câncer de mama em 23 países localizados na África subsaariana e em partes das Américas do Sul e Central ^2^ . Séries históricas de dez anos consecutivos mostraram que 8 dos 11 países da Ásia central tiveram aumento das taxas de mortalidade em mulheres com menos de 50 anos ^3^ . No Brasil, informações fornecidas pelo Departamento de Informática do Sistema Único de Saúde (Datasus) ^4^ mostram a ocorrência de 6.526 óbitos por CCU em 2018, com a faixa etária prematura (30–69 anos) respondendo por 74,0% das mortes. A maior concentração de óbitos ocorreu entre 50 e 59 anos.

O Pacto pela Saúde ^5^ é uma política pública, instituída em 2006, que se insere na sequência de políticas de apoio ao Sistema Único de Saúde (SUS). Ancora-se em três pilares (Pacto pela Vida, Pacto em Defesa do SUS e Pacto de Gestão) que visam otimizar as relações intergovernamentais em favor de melhor articulação e cooperação entre os entes federativos. É entendido como fundamental ao fortalecimento da atenção integral em saúde, com oferta e utilização de cuidados organizados por níveis de atenção ^6^ e regionalização dos serviços de saúde ^7^ .

Ainda que, em 2011, o modelo de relações intergovernamentais de consolidação do SUS tenha evoluído para compromissos firmados em contrato (Contrato Organizativo da Ação Pública – COAP), o CCU foi priorizado entre os indicadores primários do Pacto e permaneceu como alvo das autoridades de saúde nas versões subsequentes. Adicionalmente, a meta de redução de óbitos prematuros relacionadas à DCNT, que incluem as neoplasias malignas, foi também inserida no rol de diretrizes, objetivos, metas e indicadores ^8^ . Dois indicadores (razão de exames citopatológicos do colo uterino e proporção de seguimento/tratamento de lesão intraepitelial de alto grau do colo uterino) priorizados pelo Pacto visam o controle do CCU, e as notas técnicas e resultados estão disponíveis no Datasus ^4^ . Procedimentos de rastreamento e detecção precoce de câncer combinados com tratamentos efetivos repercutem em abordagem da doença em estágios clínicos mais precoces e na redução da mortalidade ^9^ . Nesse sentido, postulou-se que a mensuração da mortalidade por CCU pode fornecer um *insight* sobre a efetividade das ações induzidas pelo Pacto pela Saúde no Brasil.

Considerando que avaliações de políticas públicas podem contribuir para identificar seu impacto e, se necessário, sugerir a revisão de suas ações ^10^ , e tendo em vista que há escassez de estudos de monitoramento dos indicadores relacionados ao Pacto pela Saúde, o presente estudo teve como objetivo avaliar tendências nas taxas de mortalidade prematura por câncer de colo uterino depois da implantação do Pacto pela Saúde e de políticas dele derivadas, controlando pelo período antes da implantação do pacto, no Brasil e suas macrorregiões.

## MÉTODOS

Assumindo que as metas e os compromissos priorizados nas políticas relacionadas ao Pacto pela Saúde podem repercutir na redução das mortes prematuras e evitáveis em diversos contextos, incluindo o CCU, este estudo ecológico foi desenvolvido com uso de séries temporais interrompidas (STI) para verificar se a introdução do Pacto pela Saúde repercutiu em redução das mortes prematuras por CCU no Brasil e suas macrorregiões. O período de análise levou em consideração a dinâmica de adesão ao Pacto, pois, de acordo com a Nota Técnica n° 08/2012 ^11^ , 18,0% dos municípios brasileiros não aderiram imediatamente à política. Assim, para composição e análise da série histórica, definiram-se os períodos 1998–2006 como “pré-Pacto” e 2010–2018 como “pós-Pacto”. Os anos de 2007, 2008 e 2009 compuseram a fase de adesão e implantação do Pacto Pela Saúde.

O sítio do Datasus ^a^ foi consultado para obter informações sobre óbitos ocorridos nas faixas etárias quinquenais, de 30 a 69 anos, seguindo os passos: Informações de Saúde, Estatísticas Vitais, Mortalidade – 1996 a 2018, pela CID-10, Mortalidade Geral, Abrangência Geográfica: Brasil por Região e Unidade da Federação.

Foram consideradas as causas básicas de morte codificadas de acordo com a 10ᵃ Revisão da Classificação Estatística Internacional de Doenças e Agravos Relacionados à Saúde (CID-10), buscando os códigos C53 (neoplasia maligna do colo do útero), C54 (neoplasia maligna do corpo do útero) e C55 (neoplasia maligna do útero, porção não especificada –SOE), para posterior redistribuição.

A correção do quantitativo de óbitos por CCU foi feita seguindo dois passos. Primeiramente, foi realizada a redistribuição proporcional dos óbitos codificados como C55, conforme recomendam Antunes e Wünsch-Filho ^12^ ; para isso, óbitos codificados como SOE foram realocados proporcionalmente como C53 e C54. O segundo passo objetivou a correção dos óbitos registrados como de causa básica mal definida (CID-10 R00 a R99). Neste passo, utilizou-se um procedimento ^13^ que determina um percentual a ser aplicado aos óbitos de cada unidade de análise, por faixa etária e unidade de tempo. As equações matemáticas utilizadas nessa etapa podem ser encontradas em trabalho que corrigiu óbitos causados por câncer de mama ^14^ .

A população de mulheres por faixa etária de morte prematura (30 a 69 anos) foi obtida nos sítios do IBGE ^15^ (projeção da população de 2000 a 2060) e do Datasus ^4^ (população de 1998 e 1999). Os quantitativos foram organizados em oito faixas quinquenais (30–34, 35–39, 40–44, 45–49, 50–54, 55–59, 60–64, 65–69 anos).

Para verificar a força com que a mortalidade por CCU afeta a população de mulheres no Brasil e suas macrorregiões, primeiramente foram calculadas as taxas de mortalidade específicas por idade, para cada faixa etária e ano. O quantitativo corrigido de óbitos compôs o numerador, e a população projetada, segundo faixa etária e ano, compôs o denominador. A seguir, padronizaram-se as taxas pelo método direto, utilizando como padrão a população mundial ^16^ . Os resultados foram computados por 100 mil mulheres-ano.

### Análise de Séries Temporais Interrompidas (STI)

Foram implementadas modelagens STI e regressão segmentada ^17^^,^^18^ para verificar a tendência de mortes prematuras causadas por CCU após a introdução do Pacto pela Saúde. Foram introduzidos na análise dois novos componentes – rotulados como “mudança de nível” e “mudança de tendência” – e as variáveis resposta (taxa de mortalidade), ano (1998 a 2018) e tempo (sequência de 1 a 21). Para examinar a mudança de nível, foram atribuídos o algarismo 0 ao período “pré-Pacto” e 1 a “pós-Pacto”. Para verificar a mudança de tendência, foi considerado como 0 o período “pré-Pacto”, atribuindo-se, subsequentemente, os algarismos 1, 2, 3… a cada marco temporal do período “pós-Pacto”. Dessa forma, além da variável dependente taxa, entraram na regressão segmentada as variáveis tempo, mudança de nível e mudança de tendência.

Antecipando questões inerentes às análises paramétricas, verificou-se a existência de correlação de resíduos para *n* defasagens, por meio da estatística de Durbin-Watson e seus valores críticos ^19^ . Este método permite interpretar valores em torno de 2 como ausência de correlação serial significativa ^17^ e valor de *p* com nível alfa < 0,05 como sugestivo de resíduos autocorrelacionados. Por fim, foi feita a inspeção de gráficos de função de autocorrelação (ACF) e de função de autocorrelação parcial (PACF) para explorar visualmente a existência e a quantidade de possíveis defasagens implicadas no processo autorregressivo. A modelagem foi conduzida considerando-se critérios de parcimônia e qualidade de ajuste fornecida pelo critério de informação de Akaike (AIC), cujo valor menor sugere melhor qualidade de ajuste. A estimação de parâmetros foi feita via modelos lineares generalizados e com uso do comando *gls* e da plataforma RStudio.

### Análise de Sensibilidade

Foi conduzida análise de sensibilidade implementando a regressão segmentada sem exclusão do triênio de implantação do Pacto pela Saúde (com todos os anos). A modelagem considerou apenas dois segmentos, com o triênio de implantação primeiramente agregado ao período final, compondo os períodos pré-Pacto (1998–2006) e pós-Pacto (2007–2018). Na modelagem subsequente, o triênio de implantação foi deslocado novamente, sendo os segmentos definidos como pré-Pacto (1998–2009) e pós-Pacto (2010–2018).

### Procedimentos Éticos

O estudo segue as recomendações para pesquisa científica envolvendo seres humanos e foi conduzido com o uso de informações secundárias disponibilizadas *on-line* pelo Datasus, estando, por conseguinte, dispensado de procedimentos éticos formais.

## RESULTADOS

De 1998 a 2018 houve 102.850 óbitos por CCU (C53) no Brasil, e 77.101 (75,0%) ocorreram na faixa etária prematura (30 a 69 anos). O quantitativo de óbitos aumentou em 52,0% e em 55,2% depois das correções por SOE e por SOE e causas mal definidas, respectivamente. A maior proporção de óbitos ocorreu na região Sudeste (37,7%), seguida por Nordeste (28,0%), Sul (16,0%), Norte (10,7%) e Centro-Oeste (7,6%). No geral, os coeficientes de mortalidade prematura por CCU padronizados por idade foram altos. Apenas as regiões Sudeste (a partir de 2009) e Sul (em 2014) esboçaram estimativas menores que 10 por 100 mil ( Figura 1 ).

**Figura 1:** Tendências simples nas taxas de mortalidade prematuras (30–69 anos), padronizadas por idade atribuídas ao câncer do colo no útero no Brasil e nas macrorregiões, por 100 mil mulheres, de 1998 a 2018.

Os resultados da regressão segmentada que comparou os períodos pós-Pacto (2010–2018) e de referência (1998–2006) estão resumidos na Tabela . Foram estimados modelos exploratórios iniciais, pressupondo total independência dos dados, e modelos finais, corrigidos pela inserção de *n* termos relativos à autocorrelação serial, conforme especificado pela função de autocorrelação parcial.

**Tabela:** Efeito da introdução do Pacto pela Saúde nas taxas de mortalidade prematura por câncer do colo do útero no Brasil e nas macrorregiões: mudanças de nível e de tendências e parâmetros estimados por meio de análise de séries temporais interrompidas e por comparação dos períodos 2010–2018 e 1998–2006.

Comportamento final da mortalidade após a intervenção			Modelo exploratório inicial sem termos de autocorrelação serial				Modelo final com n termos de autocorrelação serial			
Regressores		Interpretação	Coeficiente	IC95%	Teste DWᵃ	AIC	Coeficiente	IC95%	AIC^b^	Termos AR^c^
Brasil										
	Mudança de nível	Elevação abrupta	0,48	-0,467 a 1,428	0,086	36,772	0,497	0,039 a 0,955	15,918	p = 3
	Mudança de tendência	Elevação progressiva	0,495	0,351 a 0,638			0,513	0,430 a 0,596		
	Intercepto		18,872	18,301 a 19,443			18,821	18,518 a 19,124		
	Tempo		-0,460	-0,561 a −0,358			-0,467	-0,523 a −0,411		
Norte										
	Mudança de nível	Elevação abrupta	2,522	-0,086 a 5,131	NS	65,065	2,629	0,422 a 4,837	61,234	p = 1
	Mudança de tendência	Não detectada	0,118	-0,276 a 0,513			0,128	-0,207 a 0,464		
	Intercepto		22,703	21,132 a 24,274			22,725	21,399 a 24,052		
	Tempo		-0,122	0,402 a 0,156			−0,133	0,371 a 0,104		
Nordeste										
	Mudança de nível	Redução abrupta	-1,062	-2,149 a 0,025	0,018	40,561	-0,635	-1,177 a −0,092	26,596	p = 2
	Mudança de tendência	Redução progressiva	-0,142	-0,307 a 0,022			-0,151	-0,231 a −0,071		
	Intercepto		14,494	13,839 a 15,149			14,516	14,201 a 14,833		
	Tempo		0,079	0,036 a 0,196			0,058	0,0007 a 0,116		
Sudeste										
	Mudança de nível	Não detectada	0,881	-0,122 a 1,886	0,098	38,336	0,889	-0,156 a 1,934	27,669	p = 1
	Mudança de tendência	Elevação progressiva	0,517	0,365 a 0,669			0,516	0,358 a 0,674		
	Intercepto		15,754	15,149 a 16,359			15,753	15,123 a 16,383		
	Tempo		-0,609	-0,716 a −0,502			-0,609	-0,721 a −0,497		
Sul										
	Mudança de nível	Não detectada	1,051	-0,668 a 2,770	0,098	53,394	1,059	-0,821 a 2,940	46,765	p = 1
	Mudança de tendência	Elevação progressiva	0,918	0,657 a 1,178			0,925	0,642 a 1,208		
	Intercepto		20,091	19,056 a 21,127			20,131	18,995 a 21,265		
	Tempo		-0,869	-1,053 a −0,685			-0,873	-1,073 a −0,673		
Centro-Oeste										
	Mudança de nível	Não detectada	0,994	-2,624 a 4,613	NS	74,232	0,933	-2,271 a 4,137	73,43	p = 1
	Mudança de tendência	Elevação progressiva	0,565	0,017 a 1,112			0,59	0,103 a 1,077		
	Intercepto		21,283	19,104 a 23,463			21,333	19,407 a 23,260		
	Tempo		-0,734	-1,121 a −0,346			-0,743	-1,087 a −0,398		

IC95%: intervalo de confiança de 95%.

a Teste Durbin Watson (valor de p < 0,05 sugere autocorrelação serial).

b Critério de Informação de Akaike (valores menores sugerem melhor qualidade de ajuste).

c Termos de autocorrelação definidos pela função de autocorrelação e de autocorrelação parcial.

Os dois processos de modelagem captaram resultados bastante convergentes. Especificamente, observou-se mudança de tendência, com incremento da mortalidade prematura por CCU no Brasil e nas macrorregiões Sudeste, Sul e Centro-Oeste e estabilidade na região Norte, comparando os períodos 2010–2018 e 1998–2006. Apenas o modelo final captou mudança de tendência de declínio das taxas de mortes na região Nordeste. Um particular aspecto foi a observação de mudança de nível estatisticamente significante da mortalidade prematura por CCU – que sucedeu a introdução do Pacto no Brasil – no Norte e no Nordeste. Isso ocorreu apenas no processo de estimação de parâmetros dos modelos finais, com a inserção de termos de autocorrelação serial. Embora a contribuição direta desses achados não seja tão óbvia quando se trata de políticas públicas, eles são fundamentais para predizer o impacto das intervenções considerando estimativas preditas e contrafactuais. Tomando como exemplo o Brasil como um todo e a equação “Resultado_jt_= β_o_+ β_1_* tempo_t_+ β_2_* nível_j_+ β_3_* tendência_jt_+ e_jt_”, estimaram-se valores preditos (20,042) e valores contrafactuais (10,415), verificando-se que a taxa média de mortes prematuras por CCU observada em 2018 se aproximaria de 7,38 por 100 mil caso as iniciativas que vigoraram na fase pré-Pacto seguissem em paralelo às políticas atuais ( Tabela ).

A representação pictórica das taxas relativas ao Brasil mostra que, enquanto os coeficientes da linha de base (1998–2006) experimentaram declínio visível e prolongado em efeito contrafactual, o segmento pós-Pacto delineia a mortalidade ajustada em direção oposta, estatisticamente detectada pelos coeficientes de inclinação (mudança de nível: 0,497, IC95% 0,039–0,955; e mudança de tendência: 0,513, IC95% 0,430–0,596) ( Figura 2 ).

**Figura 2:** Efeito da introdução do Pacto Pela Saúde nas taxas de mortalidade prematuras por câncer de colo uterino no Brasil, considerando os períodos 1998–2006 (pré-Pacto), 2007–2009 (introdução do Pacto) e 2010–2018 (pós-Pacto).

O conjunto de gráficos representativos das macrorregiões facilita a comparação visual ( Figura 3 ). O cenário mais promissor vem da região Nordeste. Apesar das reduções estatisticamente significativas no nível e na tendência serem ainda discretas e pouco perceptíveis visualmente, são achados que fortalecem as ações e políticas vigentes. O fato de o traçado do efeito contrafactual induzido pelas estimativas da linha de base seguir em clara ascendência dá indícios da magnitude das taxas que a região Nordeste poderia futuramente experimentar na ausência dos ganhos possibilitados pela introdução das políticas do Pacto. Em relação às demais regiões, além do incremento abrupto no nível ter sido captado pela regressão segmentada, a região Norte esboçou os piores resultados, sobretudo pela magnitude dos valores preditos e observados, pois nenhuma mudança na tendência foi delineada. Quanto às macrorregiões mais desenvolvidas do país (Sudeste, Sul e Centro-Oeste), a observação dos gráficos também sugere que o Pacto e as políticas dele derivadas ainda não repercutiram em vantagens sobre as mortes prematuras atribuídas a CCU ( Figura 3 ).

**Figura 3:** Efeito da introdução do Pacto Pela Saúde nas taxas de mortalidade prematuras por câncer de colo uterino nas macrorregiões do Brasil, considerando os períodos 1998–2006 (pré-Pacto), 2007–2009 (introdução do Pacto) e 2010–2018 (pós-Pacto).

A análise de sensibilidade não revelou divergências em relação aos resultados principais. No geral, a etapa captou mudanças de tendências na mesma direção, tanto na interrupção em 2006 quanto em 2010. A exceção foi a série segmentada em 2010 do Nordeste, que não captou o declínio progressivo da tendência detectado previamente. A mudança de nível observada no Norte e no Nordeste não foi captada em nenhum dos dois pontos de corte.

## DISCUSSÃO

Ao analisar as taxas de mortalidade prematura relacionadas a CCU, foi encontrado um cenário desafiador para o controle dessa neoplasia no Brasil. Os coeficientes padronizados tiveram valores sistematicamente elevados, acima de 10 óbitos por 100 mil mulheres, exceto na região Sudeste. Além disso, a regressão segmentada mostrou que o Pacto pela Saúde e as políticas dele derivadas ainda não repercutiram em efeitos nacionalmente favoráveis nas ações de controle do CCU e captáveis por indicadores de mortalidade prematura.

O Pacto introduziu uma novidade na gestão da saúde no Brasil, uma vez que visava a geração de resultados e o alcance de metas delineadas pelas necessidades em saúde da população. As responsabilidades entre os entes foram seladas pelo termo de compromisso de gestão, documento que perdurou de 2006 a 2012, quando ocorreu a migração para o novo formato baseado no COAP ^20^ . Em avaliação quasi-experimental, Krott e Guimarães ^20^ mostraram que. no quarto ano de adesão em nível municipal ao Pacto, começam a aparecer resultados positivos relativos à atenção básica. Estes achados mostram a propriedade de excluir da análise os períodos destinados a adesão e implementação da política.

A eliminação do CCU tornou-se prioridade mundial ^21^ , sendo oportuno implementar avaliações que possam contribuir para a compreensão do cenário real. Embora a mortalidade prematura por CCU não se encontre claramente especificada na lista de indicadores do Pacto, os objetivos, diretrizes e metas ali contidos visam a melhoria das condições de saúde da população de modo geral, além de manter seu controle entre as prioridades incluindo a redução de mortes prematuras causadas por câncer ^11^ . No contexto do CCU, os objetivos da política visaram inicialmente: (i) aumento da cobertura para 80% do exame preventivo do CCU, conforme protocolo de 2006; e (ii) incentivo para seguir e tratar lesões pré-cancerígenas, com menor dano possível e em nível ambulatorial. Os indicadores do Pacto selecionados para a cobertura (razão de exames citopatológicos do CCU em mulheres de 25 a 64 anos e a população da mesma faixa etária) e para o tratamento (percentual de seguimento e tratamento de lesões de alto grau no colo uterino) ficaram bem abaixo da meta ^4^ entre 2006 e 2012. Além disso, estudos ^22^ mais recentes que estimaram a necessidade anual de exames e procedimentos em mulheres da idade-alvo do CCU (25–64 anos) chamaram atenção para o déficit de todos os procedimentos essenciais, que variou de 7%, nas colposcopias, a 74%, nas excisões do tipo 3.

Diante desse cenário adverso, os resultados do presente estudo relativos às macrorregiões apontam também para uma realidade de pouca efetividade e grandes desafios. Até mesmo as regiões mais desenvolvidas (Sudeste e Sul) sofrem com indicadores de mortalidade prematura por CCU em elevação, contrapondo a fase pós-Pacto com o período basal. Estudos de tendência simples, com dados de óbitos do século XXI e faixas etárias mais amplas, têm apontado para a redução da mortalidade, embora isso não esteja ocorrendo homogeneamente em todas as faixas etárias ^23^ e igualmente nas grandes regiões do país ^23^ ; apesar dos esforços, estão tendendo a aumento em algumas unidades federativas ^24^^,^^25^ .

No geral, resultados de estudos de monitoramento podem induzir à revisão de políticas em andamento e auxiliar no planejamento de novas iniciativas, bem como na tomada de decisão por parte de gestores e autoridades de saúde. Por exemplo, nos Estados Unidos, a busca pela otimização de recursos destinados à redução de óbitos prematuros por CCU levou à inclusão de indicadores de monitoramento designados para medir a captação de população prioritária ^26^ . Foi estabelecido que pelo menos 20% das mulheres recém-recrutadas para testes contra o CCU deveriam estar na condição de raramente ou nunca rastreadas.

Para operacionalizar a análise do efeito do Pacto pela Saúde sobre a tendência da mortalidade prematura por CCU, este estudo foi desenvolvido usando STI e análise de regressão segmentada ^17^ . Estas técnicas são entendidas como quasi-experimentais, apropriadas para avaliações longitudinais de intervenções clínicas ^17^^,^^27^ e no âmbito da saúde pública ^18^ . Sua aplicação em pesquisa científica é oportuna no Brasil, tendo em vista que o país conta com um amplo sistema de informação em saúde, com armazenamento de dados de rotina de razoável período de tempo, além de vir implementando diversas políticas públicas voltadas à melhoria da saúde da população que podem ser mais efetivas se submetidas a avaliações concomitantes.

Um tutorial ^18^ detalha o passo-a-passo para a implementação das STI, dando ênfase à definição clara (i) da intervenção, (ii) da/s medida/s de resultado/s, (iii) dos períodos pré- e pós-intervenção, bem como (iv) do período de implantação da intervenção. Para produzir resultados robustos, Bernal e colaboradores ^18^ recomendam atenção aos potenciais fatores de confusão e às premissas relativas a análises paramétricas.

No contexto de potenciais fatores de confusão, que podem interferir no período pós-implantação da política, chama-se atenção para a Lei n° 12.732, dos 60 dias de tratamento de câncer ^28^ . Como foi regulamentada em 2012, já poderia refletir em redução das taxas de morte por CCU, fato ainda não observado neste estudo. Por outro lado, o efeito contrafactual ( Figura 2 e 3 ), delineado a partir de coeficientes ajustados desde o início da série, mostra uma tendência de redução das mortes prematuras por CCU. Estes resultados possivelmente refletem as grandes campanhas de prevenção ao CCU implementadas pelo Programa Viva Mulher ^29^ .

O presente estudo tem algumas limitações. Primeiramente, a presença de autocorrelação de resíduos pode superestimar as medidas. Lidando com isso, foi diversificada a estimação dos modelos, sendo introduzidos termos autorregressivos com defasagem diferente de 1. Embora as estimativas tenham convergido para resultados semelhantes, uma recomendação racional seria examinar tais tendências em agregados menores (unidade federativa e municípios) e verificar o que realmente está ocorrendo em termos de mortes prematuras por CCU em anos recentes.

Outra questão é que a implementação de políticas públicas acontece gradativamente, e, como esperado, parte dos municípios brasileiros não aderiu de imediato ao Pacto pela Saúde. Considerar um período de transição para implantação da política é uma recomendação ^18^ . Por isso, foram excluídos os anos de 2007, 2008 e 2009 das análises principais, acreditando, assim, ter fornecido um panorama do efeito do Pacto no comportamento dos óbitos prematuros atribuídos ao CCU capaz de induzir o desenvolvimento de novos estudos, até incluindo grupos de controle apropriados.

Concluindo, o estudo mostrou que a mortalidade prematura por CCU é alta no Brasil e suas macrorregiões. Esta série temporal interrompida não foi capaz de revelar a efetividade das políticas derivadas do Pacto pela Saúde sobre mortes prematuras por CCU no âmbito nacional e igualmente em todas as macrorregiões. No momento em que se almeja a eliminação do CCU no mundo, o panorama fornecido pela mortalidade prematura até 2018 sugere a necessidade de melhorar a efetividade das ações com medidas de incentivo para captar mulheres que estejam verdadeiramente sob risco de desenvolver o CCU e evitar óbitos de mulheres relativamente jovens no Brasil.

## Footnotes

a Ministério da Saúde. Departamento de Informática do Sistema Único de Saúde – DATASUS. Acesso em 31 mar 2020. Disponível em: http://www2.datasus.gov.br/DATASUS/index.php .
